# Neutrophil mediated smooth muscle cell loss precedes allograft vasculopathy

**DOI:** 10.1186/1749-8090-5-52

**Published:** 2010-06-22

**Authors:** Chelsey L King, Jennifer J Devitt, Timothy DG Lee, Camille L Hancock Friesen

**Affiliations:** 1 Department of Pathology, 5850 College St, Dalhousie University, Halifax, NS, B3H 1X5, Canada; 2 Department of Microbiology & Immunology, 5850 College St, Dalhousie University, Halifax, NS, B3H 1X5, Canada; 3 Department of Surgery, 5850 College St, Dalhousie University, Halifax, NS, B3H 1X5, Canada

## Abstract

**Background:**

Cardiac allograft vasculopathy (AV) is a pathological process of vascular remodeling leading to late graft loss following cardiac transplantation. While there is consensus that AV is alloimmune mediated, and evidence that the most important alloimmune target is medial smooth muscle cells (SMC), the role of the innate immune response in the initiation of this disease is still being elucidated. As ischemia reperfusion (IR) injury plays a pivotal role in the initiation of AV, we hypothesize that IR enhances the early innate response to cardiac allografts.

**Methods:**

Aortic transplants were performed between fully disparate mouse strains (C3H/HeJ and C57BL/6), in the presence of therapeutic levels of Cyclosporine A, as a model for cardiac AV. Neutrophils were depleted from some recipients using anti-PMN serum. Grafts were harvested at 1,2,3,5d and 1,2wk post-transplant. Ultrastructural integrity was examined by transmission electron microscopy. SMC and neutrophils were quantified from histological sections in a blinded manner.

**Results:**

Grafts exposed to cold ischemia, but not transplanted, showed no medial SMC loss and normal ultrastructural integrity. In comparison, allografts harvested 1d post-transplant exhibited > 90% loss of SMC (p < 0.0001). SMC partially recovered by 5d but a second loss of SMC was observed at 1wk. SMC loss at 1d and 1wk post-transplant correlated with neutrophil influx. SMC loss was significantly reduced in neutrophil depleted recipients (p < 0.01).

**Conclusions:**

These novel data show that there is extensive damage to medial SMC at 1d post-transplant. By depleting neutrophils from recipients it was demonstrated that a portion of the SMC loss was mediated by neutrophils. These results provide evidence that IR activation of early innate events contributes to the etiology of AV.

## Background

Cardiac transplantation remains the predominant treatment for patients suffering from end stage heart failure [1]. Although significant improvements in early survival post-cardiac transplantation can be attributed to new developments in immunosuppressive therapies, rates of late rejection remain unchanged, yielding only 50% graft survival at 10 years post-transplant [2]. One of the major contributing factors for late cardiac graft failure is allograft vasculopathy (AV) [3-5]. AV is a pathological diagnosis that is characterized by vascular remodelling of the graft coronary arteries which results in a neointimal lesion that eventually impairs blood flow to the heart [6,7]. Although the pathological changes of AV are well documented, the etiology remains unclear.

There is general consensus that AV is an immune mediated phenomenon [8-10]. Syngeneic transplants and transplants into immune deficient mice (such as RAG 1^-/- ^mice) fail to develop AV [11-13]. In rodent models, CD8+ T cells have been shown to be required for AV in the presence of clinically relevant levels of immunosuppression and reconstitution of immunodeficient animals with CD8+ T cells is sufficient to restore the development of AV [14]. Recent evidence [15,16] indicates that allo-antibody may play a contributory role in AV, but is not essential for its development. While few debate the critical role that T cells play in acute and chronic rejection of allografts, only recently have studies begun to acknowledge the extent to which the innate immune system regulates the potency and nature of allograft rejection [17,18].

Although there is consensus that adaptive immunity plays a pivotal role in AV, there remains controversy over the target of alloimmune damage. Some [19-21] have proposed that the damage to the endothelium initiates vasculopathy, while we [22] and others [23] have demonstrated medial damage to be more important. Reidy and colleagues used a mechanical injury model of vasculopathy and found that endothelial denudation was insufficient to induce vasculopathy [23]. However, the degree of vasculopathy correlated directly to the level of medial smooth muscle cell (SMC) loss, causing Reidy *et al*. to propose a relationship between SMC loss and neointimal vasculopathy. Nejat *et al*. [24] provided evidence that the extent of medial SMC loss correlated with developing lesion formation in a fully mismatched murine immunosuppressed transplant model. Human autopsy material from late rejecting hearts is consistent with the observations from rodents (unpublished).

Ischemia reperfusion (IR) injury is an inevitable and important insult incurred during transplantation. Extended cold ischemic times have been shown to exacerbate acute and chronic rejection making the clear link between IR and immune mediated graft damage [12,25]. An important component of IR injury induced damage occurs in the graft vasculature and may exacerbate innate immune events which impact downstream adaptive immune responses and the development of AV. Potent inflammatory chemokines, such as tumour necrosis factor (TNF) and interleukin 1 (IL-1), are expressed in syngeneic and allogeneic grafts within hours of reperfusion [26]. These, and other, chemokines promote the recruitment of neutrophils (NФ) [12,26-29]. The functional activity of these NФ and the implications of this early NФ influx to later lesion development are unclear.

## Methods

### Animals

Male, 8-10 week old C3H/HeJ (C3H;H-2^K^) and C57BL/6J (B6; H-2^b^) mice were used as donors to 8-10-week old male B6 recipients. In one experiment, male 8-9 week old C57BL/6J-Tg (ACTB-EGFP) 10sb/J (B6-GFP; H-2^b^) were used as donors to C3H/HeJ recipients. All mice were purchased from Jackson Laboratories (Bar Harbor, ME) and maintained in the Carlton Animal Care Facility, Sir Charles Tupper Medical Building, in a pathogen free environment. Food and water were given ad libitum. All animal experimentation was undertaken in compliance with the guidelines of the Canadian Council on Animal Care, under an approved animal protocol.

### Aortic Interposition Graft

Abdominal aortic segments were transplanted as we have previously described [30]. Briefly, a section of abdominal aorta approximately 1 mm in length was harvested from the donor mouse and flushed with saline solution and maintained for 20 min in cold saline before transfer into the recipient. The recipient infrarenal abdominal aorta was isolated. After proximal and distal clamping, the recipient aorta was transected and the donor aorta was interposed with proximal and distal end-to-end anastomoses using interrupted 11-0 nylon suture. The clamps were removed and blood flow was confirmed by direct inspection.

### Immunosuppression

Cyclosporine (CyA; Sandimmune iv™) was purchased from QEII Pharmacy, Halifax, NS. Aortic allograft recipient mice were treated subcutaneously with CyA 50 mg/kg/d in sterile saline for the duration of the experiment.

### Neutrophil depletion

In some experiments neutropenia was induced in B6 recipients as described [31]. Briefly 500 uL rabbit-anti-mouse PMN serum (1:10 in saline; Cedarlane; Burlington, ON) was injected intraperitoneally (IP) for three consecutive days before surgery and every day afterwards. Compared to a control experiment, this treatment resulted in > 80% depletion of thioglycollate elicited peritoneal neutrophils assessed by Geimsa stained cytospins.

### Histology

Aortas were harvested at 1d, 2d, 3d, 5d, 1wk and 2wk post-transplantation. Aortic grafts were flushed with heparinised saline and fixed in 10% formalin at 4°C for 16 h. Grafts were subsequently transferred to cold (4°C) phosphate buffered saline (PBS) for 1-2 h and then transferred into 70% ethanol until processing. Samples were embedded in paraffin and 5 μm cross sections were sectioned with a microtome. Sections were stained with Harris' Haematoxylin and 0.5% Eosin (H&E) for general histology. SMC were enumerated based on nuclear morphology, in a blinded fashion, after the staff were trained by an anatomical pathologist.

GFP aortas were harvested at 5d and 2wk post-transplant and fixed overnight in 4% paraformaldehyde in phosphate buffer followed by overnight incubation in 30% sucrose. Aortas were embedded in 10% gelatin and fixed overnight in 4% paraformalyde followed by 30% sucrose. Gelatin blocks were frozen and sectioned at 10 μm using a Leica Cryostat (Wetzlar, Germany). Sections were visualized by fluorescence microscopy on a Zeiss Fluorescent microscope using a wavelength of 450-490 nm. All images were captured with a Zeiss AxioCam camera (Carl Zeiss; Thornwood, NJ).

### Transmission Electron Microscopy (TEM)

Aortic segments were fixed with 2.5% gluteraldehyde in 0.1 M sodium cacodylate buffer and post-fixed with 1% osmium tetroxide. After washing, the segments were incubated in 0.25-0.5% uranyl acetate, rinsed and dehydrated through acetone. Segments were embedded in epon-araldite resin and sectioned using a Reichert Jung Ultracut E ultramicrotome with a diamond knife. The sections were stained with lead citrate and uranyl acetate before examination on a JOEL 1230 transmission electron microscope at an accelerating voltage of 80 kV. Digital images were captured using a CCD digital camera.

### Immunohistochemistry

Paraffin sections were deparaffinised in xylene, hydrated through an ethanol series, washed in PBS and antigen retrieval was performed. Endogenous peroxidase was quenched with a 15 min incubation in 3% hydrogen peroxide. Non-specific blocking was performed with serum specific to the secondary antibody used. The primary antibodies used were rat anti-mouse neutrophil (1:1500; Cedarlane; Burlington, ON), rat anti-mouse Ki-67 (1:1500; Dako; Mississagua, ON), and mouse anti-mouse α-actin (1:1500; Sigma; Saint Louis, MO). Primary antibody was detected with the appropriate biotinylated secondary antibody. Sections were then incubated with a peroxidase avidin/biotin complex kit (Vector Labs Inc; Burlingame, CA). One drop of prepared 3,3'-diaminobenzidine (DAB; Dako; Mississauga, ON) in 1 ml of substrate buffer was used as the chromogen. Sections were counterstained with Mayer's Haematoxylin. All enumerations were performed in a blinded fashion.

### Statistics

Data are presented as mean ± SEM for each experimental group. All statistics were performed using GraphPad Prism^® ^(GraphPad software Version 4; San Diego CA, USA). Results were analyzed by unpaired t-tests using the Welch's correction. Values of p < 0.05 were considered significant.

## Results

### Medial SMC are lost immediately post-transplantation

To examine pathological events occurring immediately post-transplantation, aortic allografts were transplanted between fully disparate strain combinations (C3H to B6) with calcineurin inhibitor (CNI) immunosuppression. This model most closely mimics the clinical situation of immunologically disparate grafts and CNI immunosuppression.

**Figure 1:**
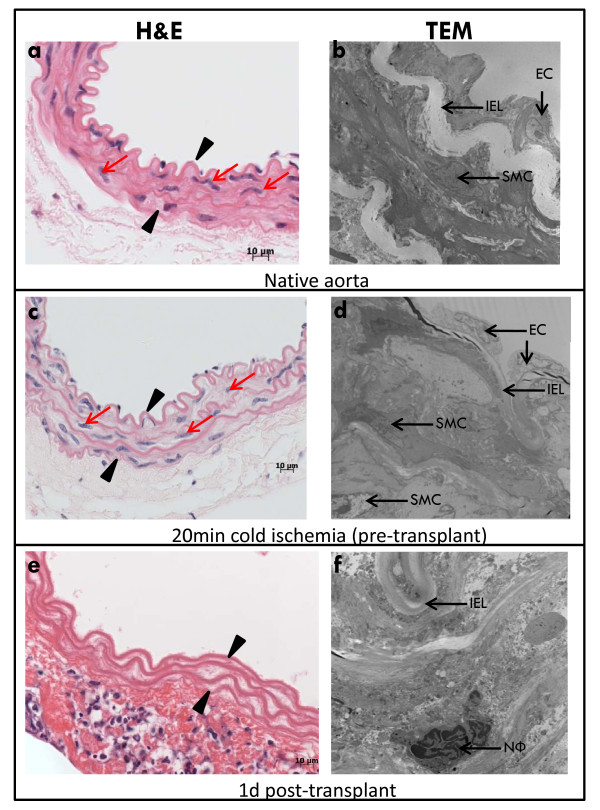
**Dramatic SMC loss occurs by 1d post transplant**. (a) and (b) native C3H aorta; (c) and (d) native C3H aorta after 20 min cold storage (pre-transplant); (e) and (f) C3H allograft transplanted into B6 recipient harvested at 1d post-transplant. Arrow heads demarcate the media. Red arrows indicate medial SMC. IEL = internal elastic lamina. SMC = smooth muscle cell. EC = endothelial cell. NФ = neutrophil.

**Figure 2:**
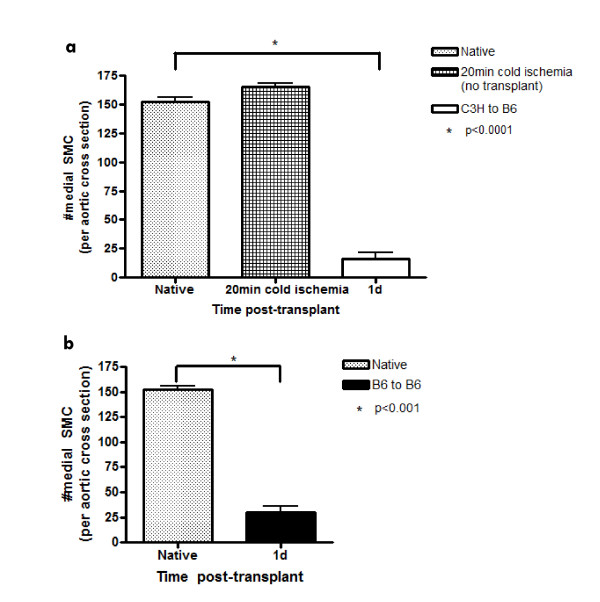
**SMC numbers are preserved following cold storage but dramatically lost at 1d post transplant**. (a) SMC counts per aortic cross section (mean ± SEM) in native C3H aorta, native C3H aorta after 20 min cold ischemia (pre-transplant) and C3H allograft transplanted into a B6 recipient at 1d post-transplant; (b) SMC counts per aortic cross section (mean ± SEM) in native C3H aorta and B6 syngeneic graft at 1d post-transplant. n = 4-6 per time point.

Figure 1 shows native aorta with substantial numbers of SMC in the media by H&E and TEM (Fig 1a,b). To examine the effect of cold ischemia on SMC we examined grafts after 20 min cold ischemia histologically and at the ultrastructural level using TEM (Fig 1c,d). Native grafts that were not exposed to cold ischemia and grafts exposed to 20 min cold ischemia (but examined pre-transplantation) had normal ultrastructural integrity. We observed well-preserved endothelium, healthy media with numerous viable SMC and normal nuclear morphology with peripheral chromatin and an intact nuclear envelope. Mitochondria showed no evidence of damage to the outer membrane and normal cristae morphology (data not shown). In stark contrast, at 1d post-transplant, endothelial cells and medial SMC were absent from the intima and media respectively (Fig 1e,f). Nuclei observed at this time point were lacking peripheral chromatin and showed extensive nuclear blebbing. These observations indicate that by 1d post-transplant, marked damage to the medial SMC had occurred. Enumeration of SMC on histological sections from native aorta, native aorta after 20 min cold ischemia (pre-transplant) and allogeneic grafts harvested at 1d post-transplant (20 min cold ischemia) demonstrated that there is no SMC loss in the grafts after 20 min cold ischemia and profound medial SMC loss (> 90% vs native aorta) at 1d post-transplant (Fig 2a). To eliminate the involvement of the adaptive immune response, we enumerated SMC on histological sections from syngeneic grafts (Fig 2b) which showed similar profound SMC loss at 1d post-transplant.

### Medial SMC repopulation of aortic grafts

**Figure 3:**
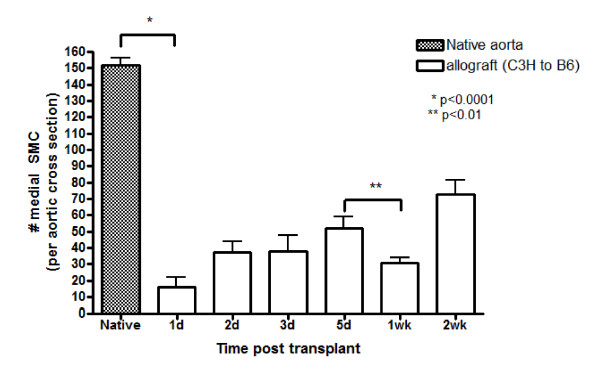
**Medial SMC numbers follow a biphasic pattern of loss and recovery**. SMC counts (mean ± SEM) per aortic cross section at 1d, 2d, 3d, 5d, 1wk and 2wk post-transplant. n = 4-6 per time point.

Having established marked medial SMC loss immediately (1d) following transplantation, we next examined whether this damage was permanent or if SMC repopulated the medial compartment. This is of significant interest because we have previously shown [24] in this model that medial SMC numbers are present at relatively normal levels at 4wk post-transplant. To elucidate the progression of SMC loss and repopulation, we enumerated SMC in the grafts at 1d, 2d, 3d, 5d, 1wk and 2wk post-transplant (Fig 3). SMC numbers began to recover by 5d post-transplant until they unexpectedly declined again at 1wk post-transplant (Fig 3). Another period of SMC recovery was observed at 2wk post-transplant (Fig 3).

**Figure 4:**
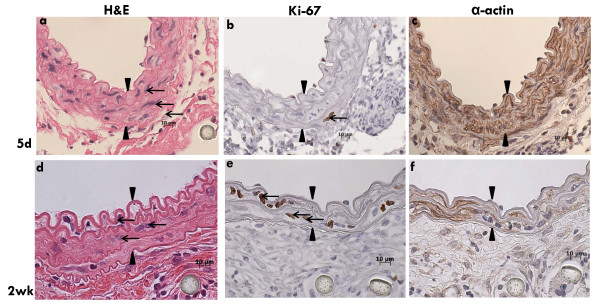
**The medial compartment is repopulated with proliferating SMC**. Serial sections of C3H allograft transplanted into B6 recipient harvested at 5d post-transplant (a) H&E; (b) immunohistochemistry for Ki-67; (c) immunohistochemistry for α-actin. Serial sections of C3H allograft transplanted into B6 recipient harvested at 2wk post-transplant (d) H&E; (e) immunohistochemistry for Ki-67; (f) immunohistochemistry for α-actin. Arrow heads demarcate the media. Arrows indicate medial SMC.

To examine the mechanisms by which SMC repopulated the medial compartment serial sections from both recovery periods (5d and 2wk) were stained with H&E, Ki-67 and α-actin. At 5d post-transplant SMC had repopulated the media (Fig 4a) and Ki-67 positive cells were observed (Fig 4b). Regions of proliferation stained positive for α-actin (Fig 4c). Results were similar for the second recovery at 2wk post-transplant (Fig 4d,e,f). These findings demonstrate the entirely novel and surprising finding of a biphasic loss and repopulation by proliferation of medial SMC immediately after transplant.

### Recovering SMC are donor in origin

**Figure 5:**
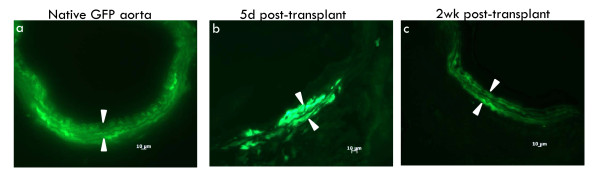
**The recovering SMC are donor in origin**. (a) native B6-GFP aorta showing fluorescing medial SMC; (b) B6-GFP allograft transplanted into C3H recipient at 5d post-transplant showing scattered, fluorescing SMC within the media indicating their donor origin; (c) B6-GFP allograft transplanted into C3H recipient at 2wk post-transplant showing well organized, fluorescing SMC within the media. Arrow heads demarcate the media. n = 3-4 per time point.

As SMC exhibited a biphasic recovery at 5d and 2wk post-transplant, we next investigated whether the SMC that repopulated the medial compartment were donor or recipient in origin. The origin of recovering SMC is an important consideration as it has been reported that late SMC loss is mediated by adaptive immunity [24]. If this is the case, then the early recovered medial SMC must be of donor origin to provide the necessary alloimmune target. To examine the origin of recovering SMC, GFP-B6 aortas were transplanted into C3H recipients in the presence of 50 mg/kg/d CyA and harvested at 5d and 2wk post-transplant. Examination of native GFP aorta (Fig 5a), allografts harvested at 5d (Fig 5b) or 2wk post-transplant (Fig 5c) showed GFP-positive cells in the media. These results confirm that the early recovering SMC are donor in origin.

### Neutrophils infiltrate the media of allografts

**Figure 6:**
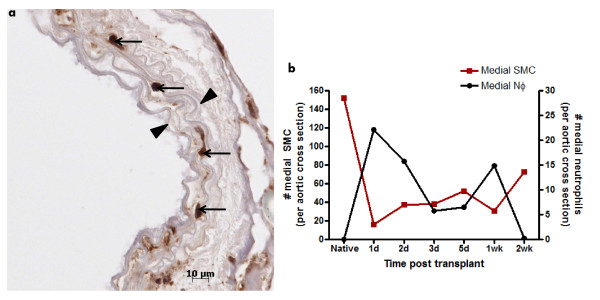
**Neutrophil infiltration is evident in the media of allografts immediately post transplant and inversely correlates with SMC loss**. (a) C3H allograft transplanted into a B6 recipient harvested at 1d post-transplant showing NФ (indicated by arrows) within the media (demarcated by arrowheads); (b) mean medial SMC (red squares) and NФ (black circle) per aortic cross-section were enumerated between 1d and 2wk post-transplant. n = 4-6 per time point.

NФ are commonly associated with ischemia reperfusion injury and their ability to cause damage and death to nearby cells is well-documented [27,32]. To investigate their role in the early events following transplantation, grafts were stained for the presence of NФ using immunohistochemistry (Fig 6a). We observed NФ infiltration in all vessel layers. NФ were enumerated in the media at 1d, 2d, 3d, 5d, 1wk and 2wk post-transplant (Fig 6b). These results demonstrate that the first peak of NФ influx into the media occurred at 1d post-transplant. After 1d post-transplant, there was a drop in NФ numbers followed by a second peak at 1wk. When the number of NФ infiltrating the media over time was compared to the level of SMC destruction, a clear inverse relationship was observed (Fig 6b). This data suggests a causative link between NФ infiltration and SMC loss in the media.

### Neutrophil depletion ameliorates SMC loss

**Figure 7:**
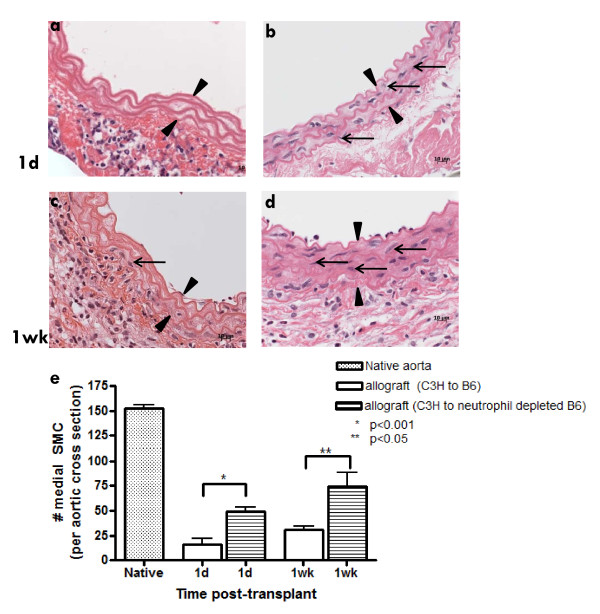
**Neutrophils mediate SMC loss**. (a) C3H allograft transplanted into a B6 recipient harvested at 1d post-transplant with dramatic loss of SMC; (b) C3H allograft transplanted into NФ depleted B6 recipient at 1d post-transplant showing partial preservation of medial SMC; (c) C3H allograft transplanted into B6 recipient at 1wk post-transplant exhibits significant SMC loss; (d) C3H allograft transplanted into NФ depleted B6 recipient at 1wk post-transplant with SMC present in the media; (e) mean ± SEM medial SMC counts for untreated control and NФ depleted recipients at 1d and 1wk post-transplant. Arrow heads demarcate the media. Arrows indicate medial SMC. n = 3-6 per time point.

Our observation of significant medial NФ influx coupled with the fact that they could be linked to medial SMC loss led us to hypothesize that NФ depletion would prevent SMC loss. B6 recipients were depleted of NФ for 3 consecutive days before surgery and daily until harvest at 1d or 1wk post-transplant. In allograft controls harvested 1d post-transplant the media was nearly devoid of SMC (Fig 7a). When recipients were depleted of NФ, there were significantly more medial SMC compared to control (Fig 7b). Similarly, at 1wk post-transplant, at the time of the second wave of SMC loss in controls (Fig 7c), NФ depletion of recipients ameliorated this SMC loss significantly (Fig 7d). SMC enumeration at 1d and 1wk post-transplant (Fig 7e) with or without prior NФ depletion of recipients confirms the results shown in the representative histological images. These data provide strong evidence to support the hypothesis that NФ contribute significantly to early medial SMC loss.

## Discussion

It is generally accepted that IR injury is a significant event in the initiation of solid organ transplant rejection [9,12,25,33]. IR injury induces a robust inflammatory response [27,34-36] which signals danger and leads to effective adaptive immune responses [27,34,36]. Necrotic injury due to ischemic damage also provides a pool of alloantigen for dendritic cell uptake and presentation to alloreactive T cells [37]. Prolonged ischemia results in more robust acute cellular rejection events in experimental models and clinical studies. Early acute cellular rejection events have been shown in a variety of studies to be an independent risk factor for the development of AV [38-42]. Likewise, studies have demonstrated that enhanced IR damage results in exacerbated late graft rejection [12,25,43]. The mechanism(s) by which IR aggravates AV may be upregulated innate immune response to the IR-injured graft, direct effects of IR on acute rejection, direct effects of IR-injury on late adaptive immune events, or a combination thereof.

Our results demonstrate the novel finding that there was a remarkable (> 90%) loss of medial SMC within 1d of transplantation. This SMC loss did not occur only during organ storage as evidenced by TEM studies which revealed preserved medial SMC in aortic grafts prior to transplantation but after exposure to cold ischemia.

Surprisingly, our study revealed that a second wave of SMC loss occurred at the 1wk time point. The second wave of medial SMC loss cannot be directly attributed to IR injury because the grafts were not subjected to any additional ischemic insult post-transplantation. Additionally, this second loss is unlikely to be mediated by T cells, as we have demonstrated that in the presence of CNI immunosuppression, T cells first infiltrate the graft at 4-5wk post-transplant [44]. There was a similar significant loss of SMC at 1d post-transplant in syngeneic transplants which highlights the role of innate immunity in this observed SMC loss. Coincident with SMC loss at 1d and 1wk post-transplant there were two waves of NФ influx into the media of allografts. NФ are the ubiquitous first responders to ischemic injury in either a syngeneic or an allogeneic milieu. NФ are recruited to transplants through chemokines secreted by the graft vasculature [26,28,29]. NФ granules contain numerous proteases and are capable of generating a variety of reactive oxygen species, thus they have been implicated as mediators of damage in models of myocardial infarction and transplantation [27,45-49] and are potentially responsible for some of the SMC damage early post transplantation. When transplant recipients were depleted of NФ, medial SMC were preserved, suggesting that NФ are responsible for some of the profound SMC loss we witnessed early after transplantation.

We have shown that SMC numbers recover with a biphasic profile, similar to the two waves of SMC loss, at 5d and 2wk post-transplant. In theory SMC recovery could be due to recipient precursor cells being recruited to fill the medial compartment. However, given that medial SMC numbers are at near normal levels by 4wk post-transplant [24] after which time they fall precipitously as a result of alloreactive CD8+ T cell mediated killing [50], it seemed more plausible that the early recovering SMC were donor in origin. By using GFP donor grafts, we established that the recovering SMC were GFP positive, indicating that they are donor in origin. Additionally, at the peak times of SMC recovery (5d and 2wk) α-actin, Ki-67 positive SMC were present in the media, further confirming that donor SMC which had been spared in the media were repopulating by proliferation. It is interesting to note that the SMC recovery in the medial compartment is donor in origin. This is in stark contrast to the neointimal lesion which we [51] and others [52] have shown to be recipient in origin.

Previous studies have implicated ischemic damage to endothelial cells as a potential initial insult in the development of AV [19,20,33,53]. Many of the murine studies have been viewed in the context of a paradigm where endothelial events were thought to lead to medial SMC de-differentiation and migration into the intimal compartment to induce the characteristic neointimal lesion of AV [8,53-55]. Newer evidence has demonstrated two important elements of this pathological process which challenge the established paradigm of the primacy of endothelial-based events in the development of AV. First, in murine models, the neointimal lesion is predominantly of recipient, not donor origin [51,52,56-59]. Second, in murine models, damage to the media appears to be more important that the endothelium in the induction of the neointimal lesion in AV [22].

The role of early SMC loss in the eventual development of AV is unclear. Our data, however, suggest that the development of AV is considerably more complex than a simple reaction to SMC loss. For instance, while there is a direct link between late medial SMC loss (5-8wk) post-transplant and neointimal lesion formation [24], there is no neointimal lesion formation after early SMC loss (1d or 1wk) post-transplant. Moreover, depletion of medial SMC by adaptive immunity late post-transplant (5-8wk) does not result in medial SMC repopulation. In contrast, there is proliferation and repopulation of donor SMC in the media which occurs early post-transplant (1d and 1wk).

These experiments were performed using CNI inhibition, the mainstay of immunosuppressive therapy post-cardiac transplant. In the clinical situation patients receive combination therapy. The impact of these additional therapies, particularly anti-proliferative therapies on SMC recovery is unknown, but of significant clinical relevance.

## Conclusion

While there is no debate that the endothelium is injured by IR and plays a role in the development of AV, emerging evidence also suggests a significant role for the medial SMC in this pathophysiology. We have shown, for the first time, early devastation of the medial compartment as a result of IR and subsequent transplantation. We have also shown that the innate immune system (NФ) is partially responsible for the early medial SMC loss in our allograft model. The interaction of the innate immune system and subsequent adaptive immune systems in the development of AV remains to be elucidated.

## List of abbreviations

AV: allograft vasculopathy; CNI: calcineurin inhibition; CyA: Cyclosporine A; IL: interleukin; IR: ischemia reperfusion; NФ: neutrophil; SMC: smooth muscle cell; TEM: transmission electron microscopy; TNF: tumour necrosis factor.

## Competing interests

The authors declare that they have no competing interests.

## Authors' contributions

CK carried out all experimental procedures with the exception of the transplants, participated in research design and helped to draft the manuscript. JD participated in the design of the study, performed all allograft harvests and helped draft the manuscript. TL and CHF conceived of the study, participated in its design and helped draft the manuscript. All authors have read and approved the final manuscript.
